# Oxidative Stability of Avocado Snacks Formulated with Olive Extract as an Active Ingredient for Novel Food Production

**DOI:** 10.3390/foods12122382

**Published:** 2023-06-15

**Authors:** Carmen Martínez, Alfonso Jiménez, Maria Carmen Garrigós, Arantzazu Valdés

**Affiliations:** Analytical Chemistry, Nutrition & Food Sciences Department, University of Alicante, P.O. Box 99, 03080 Alicante, Spain; carmen.mp@ua.es (C.M.); alfjimenez@ua.es (A.J.); mc.garrigos@ua.es (M.C.G.)

**Keywords:** avocado snacks, olive extract, oxidative stability, antioxidant performance, fatty acids, hexanal, FTIR, clean label

## Abstract

Analysis of the oxidative stability of novel avocado chips with added natural extracts was carried out with the aim of reducing the chemical additive content in their formulation. Two different natural extracts were initially evaluated and characterized: one obtained from olive pomace (OE) and other from pomegranate seed waste. OE was selected due to its better antioxidant potential according to FRAP, ABTS, and DPPH assays as well as its higher total phenolic content. The formulations used were 0, 1.5 wt.%, and 3 wt.% of OE. A gradual disappearance of the band found around 3009 cm^−1^ and related to unsaturated fatty acids was observed in the control sample in contrast to formulations with added OE. The band observed near 3299 cm^−1^ widened and intensified with time due to the oxidation degree of samples, with this effect being higher in the control chips. The observed changes in fatty acid and hexanal content with storage time underlined the higher extent of oxidation in the control samples. This fact could suggest an antioxidant protectant action of OE in avocado chips during thermal treatment, which was attributed to the presence of phenolic compounds. The obtained chips incorporating OE represent a viable option for the development of a natural, healthy, and clean-label avocado snack at competitive cost and with low environmental impacts.

## 1. Introduction

In recent times,, there has been an increasing interest in the consuming healthier natural products with functional properties and with a positive impact on human health [1,2]. One of the most promising dietary strategies is the use of natural ingredients for the development of novel foods as there has been an increase of prevalence of different diseases as a consequence of lifestyle changes [3]. The global consumption of ready-to-eat food snacks has grown in the past two decades because of radical changes in eating habits [4]. Snack food can be defined as a small portion of food eaten between regular meals [5]. According to the Snack Food Global Market Report 2023 [6], the global snack food market grew from USD 237.65 billion in 2022 to USD 256.5 billion in 2023 at a compound annual growth rate (CAGR) of 7.9%, with a post-pandemic scenario that it has promoted this situation [7,8]. In this context, the Asia-Pacific region held the largest share of the snack food market in 2022, and North America the second-largest share. The snack food market comprises sales of snacks created through different industrial processes. These snacks include nuts, seeds, and grains such as salted, dried, roasted, buttered, or fried; chips of potato and corn; and popped popcorn.

In recent years, there has been a growing concern about the need to reduce agro-industrial waste in the environment. In this context, the bioeconomy, which involves mobilizing biomass resources, is the key strategic innovation pillar in the European Union [9,10,11]. In a recent report published in 2018 [12], the European Commission estimated a total average agricultural biomass production in the EU for the period 2006–2015 of 956 million tons of dry matter per year (Mt/a), with being 514 Mt (54%) of this being primary products (biomass produced as grains, fruits, roots, tubers, etc.), whereas the other 442 Mt of biomass (46%) was secondary products such as dry biomass from leaves and stems (residue production).

Olives are a widely consumed fruit with high nutritional value. They are also used to produce extra virgin olive oil, a highly recognized product. The worldwide olive production in 2021 showed an increase from 21 million tons (Mt) in 2017 to around 23 Mt in 2021. Spain was the main producer with 35.8% of the total production followed by Italy (9.8%), Turkey (7.5%), Morocco (6.9%), and Portugal (6.0%) [13] The olive oil industry generates large amounts of waste, mainly olive pomace (skins, bones, and pulp) and olive-mill wastewater. These residues contain high contents of lipids, organic acids, and phenolic compounds [14]. Olive pomace is the major by-product accounting for up to 80–95 g 100 g^−1^ of semisolid mass in the olive oil industry, with a production estimation higher than 2.8 million tons/year worldwide. This residue is mainly composed of fatty acids, proteins, and polysaccharides, as well as polyphenols and pigments with antioxidant activity [15,16]. Among these compounds, this residue is rich in hydroxytyrosol, caffeic acid, oleuropein, vanillic acid, elenolic acid, rutin, catechol, p-coumaric acid, and verbascoside. For this reason, the valorization of this residue through the extraction of target components with phytochemical activity, such as polyphenols, has been extensively reported with applications in cosmetic, pharmaceutical, and food industries [17,18]. This by-product has been reported to be an effective antioxidant and antimicrobial active additive to be used as a potential substitute of sulfur dioxide in winemaking and for the development of active packaging materials [19].

Pomegranate (*Punica granatum* L.) fruit has been underlined in the literature with promising activity against inflammatory and chronic diseases [20,21,22]. The main production of this fruit is located in the Valencian Community, Spain, with a total annual production of 50,000 tons [23]. The non-edible arils, seeds, and rings have been reported as showing noticeable contents of isolariciresinol, from 5.0 to 13.6 mg kg^−1^, and hydrolysable tannins, from 32 to 263 g kg^−1^ [21]. Until now, the use of these residues has been investigated for film formulations [24] and the bioactive properties of extracts from pomegranate peel and seeds were also recently reported [25,26,27].

Today, plant-based foods, characterized for having a cellular structure, are widely recommended to be the cornerstone of a healthy diet. This group includes vegetables and fruits, as well as legumes and nuts. Avocado is a fruit that contains a fat fraction of 15 wt.%. Up to 71 wt.% is based on monounsaturated fatty acids [28] and there is also a high concentration of bioactive compounds, such as vitamins B, E, and C; dietary fiber; pigments; lutein; and phenolic compounds—showing its great potential in the development of healthy snacks [29]. However, the high content in unsaturated fatty acids makes these snacks prone to lipid oxidation processes [30]. On the other hand, although ready-to-eat snacks offer considerable market potential, they are perceived as unhealthy products due to their high energetic value, their salt and fat content, and the presence of trans-saturated fatty acids. Some studies reported in the literature have tested the feasibility of different drying treatments to monitor the modifications in physicochemical parameters of vegetable-based chips such as potato [31,32], pumpkin [33], jujube [34], or apples [35]. However, the addition of natural antioxidant extracts based on agricultural byproducts as a natural ingredient has not been extensively reported. Commonly, to improve the oxidative stability of food, it has been industrially treated with the addition of synthetic additives, increasing the shelf life, or improving the sensorial perception through a specific flavor. However, there have been some reports underlining the potential risks associated with the consumption of foods with synthetic antioxidants [36]. Nowadays, there is an increasing trend towards the addition of natural antioxidants extracted from animal or plant sources as preservatives and healthier alternatives to synthetic ones for clean-label foods. Thus, the search for new ingredients that avoid the use of chemical additives is a promising direction for food industries [37]. The aim of this work was to study the oxidative stability of novel avocado chips with an added functional natural extract, at different loadings, to reduce the content of chemical additives in their formulation. This represents a viable option for the development of natural, healthy, and clean-label avocado snacks at competitive cost, as well as for reducing environmental impacts. For this purpose, extracts obtained from olive pomace and pomegranate seed wastes were characterized and considered.

## 2. Materials and Methods

### 2.1. Materials

Two naturals extracts kindly provided by Probeltebio (Murcia, Spain) were used in the present study. One was an olive extract (OE) from Spanish olive fruit (*Olea europaea)* by-products. It was obtained after water extraction standardized with up to 40% wt.% of hydroxytyrosol. The other was an extract obtained after pressing the arils and seeds of the pomegranate fruit (*Punica granatum* L.) (PS) collected as residues from the industrial juice production. Avocado samples used to develop chips belonged to the Hass variety and were purchased in a local supermarket. Fruits were stored at 4 ± 1 °C under air atmosphere.

Sigma-Aldrich provided the following reagents: the fatty acid methyl esters standards, hexanal and 4-methyl-2-pentanone and the reagents for the antioxidant activity and total phenolic content measurements (DPPH (2,2-Diphenyl-1-picrylhydrazyl) reagent and ABTS (2, 2′-Azino-bis (3-Ethylbenzothiazoline-6-sulfonic acid)) diammonium salt, TPTZ (2,4,6-Tripyridyl-s-triazine), potassium persulfate, Folin−Ciocalteu reagent (2 N), sodium acetate trihydrate, Trolox, aluminum chloride and sodium nitrite). On the other hand, Panreac (Barcelona, Spain) supplied the sodium carbonate, sodium hydroxide, calcium chloride, ethanol, and iron trichloride.

### 2.2. Antioxidant Activity

DPPH radical scavenging assay, ABTS free-radical scavenging assay and ferric reducing antioxidant power (FRAP) were used to study the antioxidant activity of the extracts, as described elsewhere [21]. In this study, 100 mg kg^−1^ was used as initial concentration of extracts diluted in distilled water and Trolox was used as reference standard. All methods were carried out in triplicate.

### 2.3. Total Phenolic Content

The Folin−Ciocalteu colorimetric method was used to determine the total phenolic content of extracts. A Biomate-3 UV/VIS spectrophotometer (Thermospectronic, Mobile, AL, USA) was used in this work [38]. The following method was followed: 250 μL of initial extract at concentration of 100 mg kg^−1^ was mixed with 250 μL of Folin–Ciocalteu reagent and 3500 mL of distilled water and incubated, protected from light, for 3 min. Then, 1 mL of Na_2_CO_3_ solution at concentration of 20 wt.% was added. After 40 min at 40 °C in the dark, the absorbance at 750 nm was measured. The blank used was deionized water. Trolox was used as reference standard (50 to 500 mg kg^−1^).

### 2.4. Chip Preparation and Shelf-Life Study

**Figure 1:**
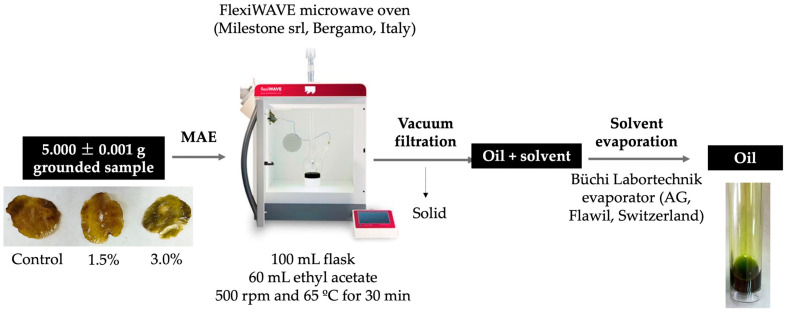
Microwave-assisted extraction procedure followed in the present study.

A vegetable-based non-extruded snack was proposed in the present work based on avocado as matrix. A dehydration treatment was selected in order to make the avocado fruit more shelf-stable, without requiring any other preparation steps (e.g., washing, peeling, or seed removal) [5]. Avocado-based chips with and without the active extract were prepared as follows. Before analysis, avocado fruits were crushed with a domestic blender for 10 s in order to homogenize them and reduce their size. Then, 8.000 ± 0.001 g of crushed avocado pulp, manually formed into a dough, was covered with vegetal paper and allowed to stand at 40 °C for 24 h in an oven (Memmert GmbH, Schwabach, Germany). Three formulations were proposed in this work by adding 0, 1.5 wt.%, and 3.0 wt.% of active extract. Figure 1 shows the final product of formulations. All samples were kept inside plastic bags in air atmosphere at room temperature (20 ± 3 °C) and protected from light. Three different times were studied, in triplicate, to evaluate the oxidative stability of avocado chips: 0, 6, and 14 days.

### 2.5. ATR-FTIR Spectroscopy Analysis

A Bruker Analitik IFS 66 FTIR spectrometer (Ettlingen, Karlsruhe, Germany) was used to collect the FTIR spectra of samples. First, the grounded and homogenized chips were directly put on the instrument and the spectra were recorded from 4000 to 400 cm^−1^, using 64 scans and 4 cm^−1^ resolution. All spectra were corrected against the background spectrum of air.

### 2.6. Fatty Acid Composition

#### 2.6.1. Microwave-Assisted Extraction (MAE) Process

The oil extraction of chips was previously necessary for the quantification of the major fatty acids. In this work, the use of a microwave-assisted extraction system following the procedure described in Figure 1 was proposed. Ground avocado chips were extracted into a 1000 mL flask with 60 mL of ethyl acetate at 65 °C for 30 min under stirring. After extraction, the liquid phase (solvent and the oil) was obtained by vacuum filtration, and distilled with a rotary evaporator at 50 °C under reduced pressure until the weight of oil remained constant.

In this work a greener solvent to extract the oil from avocado by using MAE was proposed due to its relatively less toxic nature than the more commonly used solvents such as hexane, petroleum ether, or methanol [39,40]. Previous tests were carried out to select the use of ethanol or ethyl acetate as a solvent extraction during the MAE process under the conditions described in Figure 1. The amount of extracted oil was quantified by gravimetry and Equation (1) was used to obtained the extraction yield of oil:

(1) $$Extraction\;yield\;of\;oil\left(\%\right)=\left(\frac{mass\;of\;extracted\;oil}{mass\;of\;dried\;material}\right)\times 100$$

#### 2.6.2. Analysis of Major Fatty Acid Composition by GC–MS

Methylation of fatty acids present in oil samples was carried out as described elsewhere [41]. Analysis of FAMEs was performed by using an Agilent 7820A GC System (Palo Alto, CA, USA) equipped with a FID detector. A TR-CN100 column (60 m × 0.25 mm × 0.2 µm; Teknokroma, Barcelona, Spain) was used. The GC oven was programmed from 120 to 250 °C (hold 15 min) at 5 °C min^−1^. Helium was used as carrier gas (1 mL min^−1^) and 1 µL was injected in the split mode (1:75). The tridecanoic acid methyl ester (700 mg kg^−1^) was used as internal standard whereas analytical standards were used for external calibration.

### 2.7. Hexanal Quantification

A headspace-solid-phase microextraction (HS-SPME) followed by GC–FID analysis (Agilent 7820A GC System, Palo Alto, CA, USA) was proposed in this work for the quantification of the hexanal content of chips [42]. First, 1.00 ± 0.01 g of grounded chips was prepared and then 2 mL of saturated NaCl and 20 µL of the internal standard (4-methyl-2-pentanone, 8 mg kg^−1^) was added into the SPME vial with a micro-stirring bar. In accordance with the literature, a DVB/CAR/PDMS SPME fiber for the adsorption of polar and nonpolar volatile compounds was used (Supelco, Bellefonte, PA, USA) [43]. The sample vial was placed in a water bath at 50 °C with a stirring of 500 rpm. First, 10 min of equilibration under these conditions was required. Then, the extraction was carried out during 30 min in which the fiber was exposed before its desorption during 12 min into the GC injector port at 250 °C. The splitless mode was selected in this step. The GC–FID equipment was an Agilent 7820 AGC System (Palo Alto, CA, USA) equipped with a SPB-5 column programmed in two ramps: (a) from 50 °C to 70 °C (hold 1 min) at 5 °C min^−1^ and (b) from 70 °C to 200 °C (hold 10 min) at 35 °C min^−1^. The carrier gas was helium at 1 mL min^−1^ and the FID temperature was 300 °C. Working solutions obtained from an hexanal stock prepared at 30 mg kg^−1^ in distilled water were used for the external calibration.

### 2.8. Statistical Analysis

Statistical analysis of experimental data was performed with SPSS commercial software (Version 15.0, Chicago, IL, USA). ANOVA and Tukey test were assessed for *p* < 0.05.

## 3. Results and Discussion

### 3.1. Antioxidant Activity and TPC of Active Extracts

In order to select the active extract with the highest antioxidant properties to be used as the active additive in the preparation of avocado chips, the antioxidant activity and TPC of the two studied extracts, OE and PS, were evaluated. The obtained results are shown in Table 1.

Both studied extracts showed an adequate antioxidant content according to the literature, between 56–71 mg Trolox g^−1^ extract, and TPC values ranging 36–53 mg Trolox g^−1^ extract [44,45]. OE was mainly composed of hydroxytyrosol whereas PS was rich in punicalagin, which is the main ellagitannin present in pomegranate fruit [46]. The edible part of pomegranate fruit corresponds to the arils which constitute 52 wt.% of the total weight of the fruit, comprising 78 wt.% juice and 22 wt.% seeds of its weight [47]. Phytochemically speaking, pomegranate arils and seeds are characterized by the presence of anthocyanins (glycosides made up of aglycones, the most common of which are delphinidin, pelargonidin, peonidin, petunidin, cyanidin, and malvidin, among others) and phenolic acids (ellagic acid, gallic acid, chlorogenic acid, caffeic acid, and p-coumaric acid). Both arils and seeds have been demonstrated to have important activities such as hypolipidemic activity, and antimutagenic, antioxidant, apoptotic, and antimicrobial potential [46,48,49,50].

All studied antioxidant methods, except ABTS, showed statistically significant differences in results between OE and PS. OE showed higher antioxidant activity and total phenolic content. This behavior was also supported by IC_50_ values obtained for both extracts by DPPH assay, since a lower IC_50_ value was obtained for OE (0.59 ± 0.05 µg Trolox g^−1^ extract) compared to PS (0.66 ± 0.03 µg Trolox g^−1^ extract). OE is a natural extract obtained from the fruit of the olive tree (*Olea europaea* L.) cultivated in the Mediterranean region of Spain through sustainable agriculture and extracted with a patented process which maintains the natural profile of the fruit. The olive pomace is rich in hydroxytyrosol, but also has a significant content in phenolic acids (caffeic acid, p-coumaric acid, vanillic acid, cinnamic acid, ferulic acid, gallic acid, and syringic acid), secoiridoids (oleuropein, tyrosol, verbascoside), and flavonoids (luteolin, hesperidin, quercetin, apigenin, among others) [51]. Currently, there is much research supporting the beneficial health effects of olive oil by-products. A search in Scopus using the terms “olive by-products AND health” or “olive pomace AND health” produced 176 and 129 results, respectively, (updated on 21 April 2023). The beneficial properties of olive pomace have been recently reported, including an improvement in the blood lipid profile [52], enhancement of cardiometabolic status, with a potentially positive effect on the vascular tone [53], and improvement in neurodegenerative disorders [54]. Regarding the food industry, the efficacy of adding bioactive compounds from olive pomace in food packaging applications was also reported [55]. Among olive pomace phenolics, hydroxytyrosol is known to exert advanced antiradical properties similar to vitamins E and C [51]. As a result, OE was selected in this work as active additive to be used for the processing of avocado-chip snacks since it is generally recognized as having safe (GRAS) status (by the Food and Drug Administration (FDA) in the United States) [56]. An initial characterization of the OE was previously published and is detailed elsewhere in which the addition of this extract was evaluated as an active antioxidant additive in corn starch-based films [22].

### 3.2. Oxidative Stability Study of Packaged Avocado Chips

#### 3.2.1. Structural Characterization by ATR-FTIR

**Figure 2:**
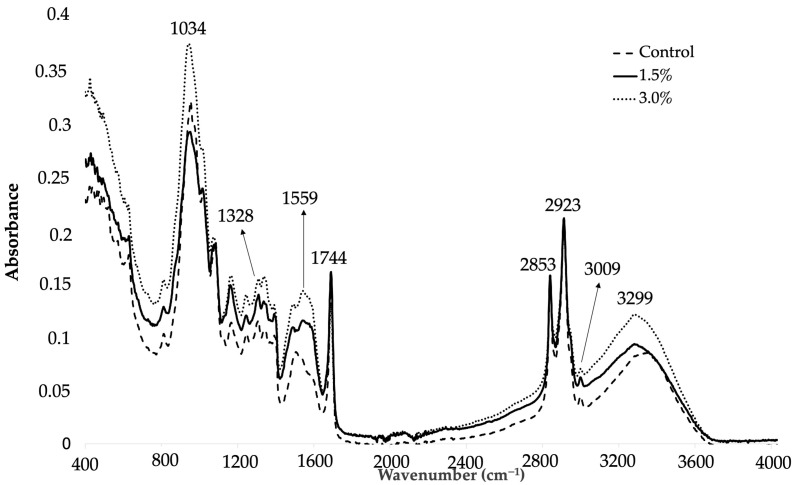
FTIR spectra of avocado-chip formulations (0, 1.5 wt.%, and 3 wt.% of OE) at day 0 of study.

Figure 2 shows the ATR-FTIR spectra of the studied avocado-chip formulations (0, 1.5 wt.%, and 3 wt.% of OE) obtained at day 0 of the study, showing the characteristic bands of carbohydrate, fat, and protein fractions of avocado composition (Table 2). Avocado pulp is a good source of various nutrients, with an average content of 15 wt.% fat, 9 wt.% carbohydrate, 6 wt.% g fiber, and 2 wt.% protein, in 100 g of fresh pulp [57,58].

Statistically significant differences (*p* < 0.05) were found for some bands in the studied formulations with time, considering wavenumber and absorbance values (Table 3). Seven parameters related to protein fraction (wavenumber near 1559 cm^−1^), lipids (wavenumber near 2853 and 2953 cm^−1^ and absorbance values of the bands near 2923 and 3009 cm^−1^), and hydroperoxides, amylose, amylopectin, and amide A (wavenumber and absorbance values of the band near 3299 cm^−1^) showed significant differences at 0, 6, and 14 days of oxidative treatment. The presence of the band near 1559 cm^−1^ was due to the bending vibration of N-H bonds, which are typical of all proteins. The wavelength of this band slightly decreased with time in all samples. As was expected, noticeable differences were observed between samples with time for the bands linked to the fat fraction. Avocado fruit is rich in oleic (56 wt.%), palmitic (21 wt.%), linoleic (14 wt.%), and palmitoleic (9 wt.%) acids [61,62]. The absorptions of the bands appearing at 2923 and 2853 cm^−1^ were attributed to tensile vibrations of CH_2_ for asymmetric and symmetric vibrations, respectively. High-frequency values in this absorption range indicate a sample rich in unsaturated and polyunsaturated acids [60]. The stretching vibration of CH cis-olefinic groups appeared around 3009 cm^−1^. The isomerization, cis to trans, of double bonds of unsaturated fatty acids has been reported in oxidative treatment progresses [63]. Therefore, a gradual disappearance of this band was expected with time. In this study, the absorbance of this band in the control sample decreased with time in contrast to formulations with 1.5 wt.% and 3 wt.% of the extract. This fact could be related with the lower oxidative degree of samples with OE in their composition. Finally, wavenumber and absorbance values of the band observed near 3299 cm^−1^ also showed statistically significant differences between samples at days 0 and 6. The protein fraction and carbohydrate constituents such as fiber, hemicellulose, and starch are significant compounds present in the seed but not in the pulp of avocado [61,64]. Thus, it can be supposed that this band widened and intensified with time due to the oxidation degree of the samples. Some authors have underlined that increases observed in this band may be due to hydroperoxide formation which generated occurs during the first stage of the oxidation process [43]. At the end of the shelf-life study, all samples showed similar absorbance values for band observed near 3299 cm^−1^, which could be related with the increased oxidation of all studied samples with time due to their degradation.

#### 3.2.2. Fatty Acid Composition

##### Selection of Solvent in MAE

The effect of different solvents on yield extraction of oil by MAE were studied. Ethanol, ethyl acetate, and the mixture of both (1:1, *v*/*v*) was chosen as potential candidate for greener extraction of the avocado-chip oil. The highest extraction yield was obtained for ethyl acetate followed by the mixture and, finally, the ethanol solvent with extraction yield values of 50 ± 4, 38 ± 2 and 19 ± 1%, respectively. The reason for this difference was mainly that the type of solvent plays an important role in MAE. Generally, the choice of solvent in MAE should be based on its solubility of the target compound [40,65]. Ethanol has more polarity with limited solubility of the oil whereas ethyl acetate has a better solubility for oil due to its lower polarity. Thus, the ethyl acetate was finally chosen for the oil extraction of avocado chips by the MAE process.

##### Analysis of Major Fatty Acid Composition by GC–MS

In this work, the major fatty acids of avocado were determined. According to the literature, oleic, palmitic, linoleic, and palmitoleic are the predominant fatty acids in avocado pulp [58,61,66]. The initial content (day 0) of monounsaturated oleic fatty acid (C18:1), linoleic (C18:2), and linolenic (C18:3) polyunsaturated fatty acids and saturated palmitic acid (C16:0) in the studied samples were (Table 4): 55.3, 27.2, 16.6, and 1.2 wt.%, respectively, for the control sample; 56.5, 27.4, 14.9, and 1.2 wt.%, respectively, for chips incorporating 1.5 wt.% of OE; and 56.4, 27.6, 14.8, and 1.2 wt.%, respectively, for formulations with 3 wt.% of added OE. Different fatty acid compositions of Hass avocado pulp reported previously in the literature are in line with these results [61,62,66,67].

**Figure 3:**
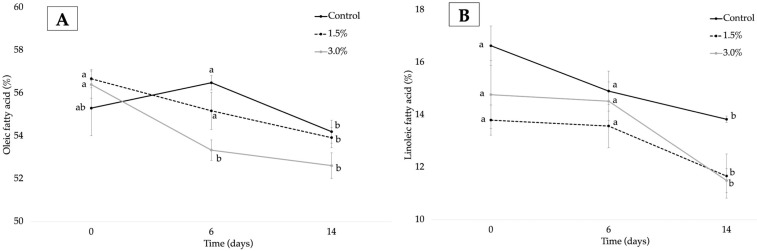
Oleic (**A**) and linoleic (**B**) fatty acid compositions for control, 1.5 wt.%, and 3 wt.% samples at 0, 6, and 14 days (n ± SD; n = 3). Different letters for each compound within the formulation indicate statistically significant different values (*p* < 0.05).

**Figure 4:**
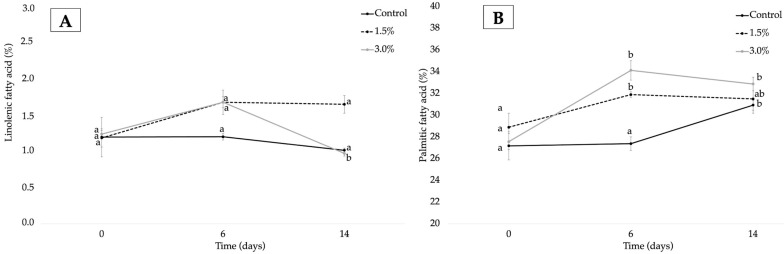
Linolenic (**A**) and palmitic (**B**) fatty acid composition for control, 1.5 wt.% and 3 wt.% samples at 0, 6, and 14 days (n ± SD; n = 3). Different letters for each compound within the formulation indicate statistically significant different values (*p* < 0.05).

It is well known that the oxidative rate of lipids is related to the ratio of poly- and mono-unsaturated fatty acids [68,69,70]. Under the proposed treatment, a general decrease in all studied fatty acids with time was observed for the control and formulations of OE, except for the saturated palmitic fatty acid. These results are in line with data previously reported for different foods with high fat content [43,68]. In relative terms, the reduction in fatty acid content for the control sample during storage followed the order linoleic (16.7 wt.%) > linolenic (15.2 wt.%) > oleic (2.0 wt.%) acids, whereas palmitic acid increased with time (12.1 wt.%). This fact could be explained by considering the number of double bonds in the molecule. According to the literature, the oxidation rate of fatty acids increases with the number of these double bonds because it is easy to remove the hydrogen atom attached to the carbon between two double bonds [71,72]. Chips with added OE showed a different behavior with time, underlining different oxidative stages in samples. In general, as OE increased, a higher decrease in oleic fatty acid content (Figure 3A) was observed in formulations incorporating 1.5 wt.% and 3 wt.% of the extract (4.9 wt.% and 6.7 wt.%, respectively), whereas a higher increase in palmitic acid content was observed for chips with 3 wt.% of OE (16.1 wt.%) (Figure 4B). Regarding linoleic fatty acid (Figure 3B), a noticeable decrease in this fatty acid was observed between 0 and 14 days of storage for the formulation with 3.0 wt.% (22.2 wt.%) followed by chips with 1.5 wt.% and the control with 15.4 and 16.6 wt.%. This different behavior was also observed for linolenic acid (Figure 4A), showing no statistical differences between control and 1.5 wt.% chips whereas a decrease was observed in formulations with 3.0 wt.% from 6 to 14 days of storage.

According to Chen et al. [73], changes observed in (C18:2/C16:0) ratio can also be used to assess the oxidative degradation of fats, with a greater reduction in this ratio indicative of a higher deterioration of polyunsaturated fatty acids [62,70]. At day 0 of storage (Table 4), control chips showed the highest ratio compared to samples with added extract. This fact could indicate a role of OE as a protectant agent against oxidation processes. This ratio significantly decreased with storage time from 0 to 14 days, with the lowest decrease in control chips (up to 27.20 wt.%) compared to formulations with added OE, which showed a decrease of 22.55 wt.% and 34.82 wt.% for chips with 1.5 wt.% and 3 wt.% of the extract, respectively. These differences were evident after 6 days of storage, with the control sample having the highest oxidation followed by the chip incorporating 1.5 wt.% and 3 wt.% of OE; the latter being the more resistant to degradation. Thus, it could be stated that control samples showed a higher extent of oxidation due to more evident fatty acid changes with time compared to that observed for samples with added OE [70]. This fact could be related to the positive antioxidant activity found for OE in this study, as was previously detailed.

#### 3.2.3. Hexanal Content

Fatty acids are precursors of aroma volatile compounds [74], which have a well-established role in determining the characteristic flavor of a wide variety of food products [67]. Lipid oxidation processes (auto-oxidation and enzymatic) result in rancidity, which can be defined as the formation of off-flavors and odors from lipids. Hydroperoxides formed during the oxidation of linoleic acid, the most abundant and oxidation-susceptible fatty acid in avocado, quickly break down to produce many secondary compounds causing rancid odor and taste, such as aldehydes and ketones [75]. Among all volatiles reported for avocado samples, hexanal compounds have been described some of the main compounds providing a particular ‘grassy aroma’ with a low aroma threshold [76,77]. The 6-carbon aldehyde, hexanal, which is produced from the degradation of linoleic and linolenic acids, has also been reported to be inherent of food with high fat content [78,79,80]. In the present study, hexanal was quantified as an indicator of rancidity in avocado chips as, according to other studies, it has a very good correlation with sensory evaluations of lipid oxidation, [70,75,77,78].

**Figure 5:**
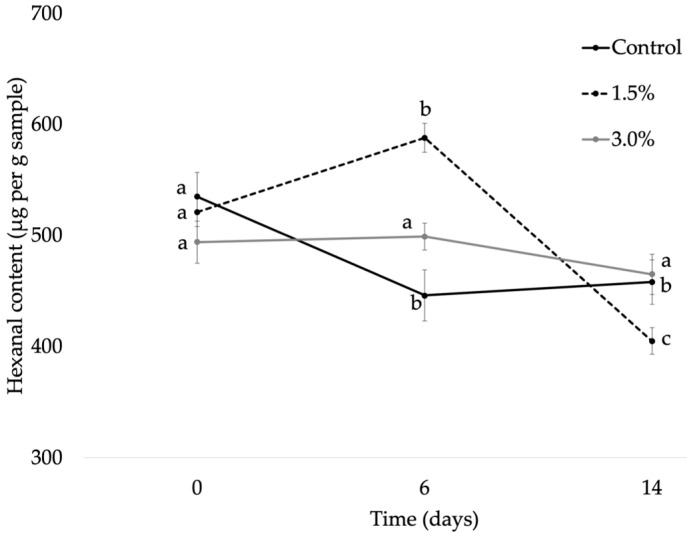
Hexanal average content (µg kg^−1^; n ± SD, n = 3) for control, 1.5 wt.%, and 3 wt.% samples at 0, 6, and 14 days. Different letters for each sample within the formulation indicate statistically significant different values (*p* < 0.05).

Figure 5 shows an increase in hexanal content between 0 and 6 days, because of sample oxidation, for samples with OE. The formulation with 1.5 wt.% of OE showed noticeable increase of this aldehyde in contrast to the rest of studied formulations. A decrease in the hexanal was observed between 6 and 14 days of study underlying the depletion of headspace oxygen in the closed vial where sample was contained. This fact has been explained in literature as being due to the reaction between the oxygen in the headspace and the unsaturated fatty acids of samples [68]. It is interesting to underline that a constant decrease was observed in control samples from the beginning of storage. This fact could be related to a higher extent of oxidation in these samples in contrast to the other ones. According to Galvao et al. [77], hexanal levels are generally lower in avocado due to the presence of a high oleic acid content, in agreement which our results. At day 0, a higher oleic acid content was found for chips with 3 wt.% added, followed by 1.5 wt.%, and the sample without the extract was less rich in this fatty acid. This fact could suggest an antioxidant protectant action of OE in avocado chips during the thermal treatment in the oven, which could be attributed to the antioxidant capacity of phenol compounds present in the studied extract [75]. After 6 days of storage, the formulation with 3 wt.% added showed higher content in oleic fatty acid compared to the 1.5 wt.% formulation, in line with the hexanal results. Therefore, the concentration of the studied extract directly affected the oxidative degradation of avocado chips.

## 4. Conclusions

The present work allowed us to increase the scientific knowledge about chemical changes occurring during the storage of avocado chips with the added natural antioxidant extract, OE, obtained from Spanish olive fruit (*Olea europaea*) by-products, and processed following a dehydration process in the oven at moderate temperatures. The addition of OE, as a strategy to improve the oxidative stability of the developed products, had different effects on the response variables evaluated in this study. In general, the formulation with 3 wt.% of added OE showed the best results, with no pronounced degradation process in these samples. Control chips without the addition of OE were less stable to the oxidative treatment. The elucidation of the degradation mechanism of fatty acids was linked to the production of hexanal volatile compounds and FTIR results, helping researchers to understand how the different technological practices available during avocado thermal processing (e.g., dehydration, frying, and roasting) could affect the overall oxidative degradation of ‘Hass’ avocado chips. Further work will be needed to evaluate the sensorial behavior of the developed chips to improve the functional properties of the obtained formulations and their possible commercialization in the real market.

## Figures and Tables

**Table 1:** Antioxidant activity and TPC results obtained for the studied active extracts (n ± SD; n = 3).

Property	PS	OE
IC_50_ (µg Trolox g^−1^ extract)	0.66 ± 0.03 ^a^	0.59 ± 0.05 ^b^
FRAP (mg Trolox g^−1^ extract)	58 ± 1 ^a^	71 ± 8 ^b^
ABTS (mg Trolox g^−1^ extract)	67 ± 9 ^a^	56 ± 6 ^a^
TPC (mg Trolox g^−1^ extract)	36 ± 1 ^a^	53 ± 1 ^b^

Different superscripts (^a,b^) within the same row and parameter indicate statistically significant different values (*p* < 0.05). ABTS, 2, 2-Azino-bis (3-ethylbenzothiazoline-6-sulfonic acid); FRAP, ferric reducing antioxidant power; IC_50_,: extract concentration required to cause 50% reduction in the initial DPPH concentration.

**Table 2:** Characteristic FTIR peaks of avocado-chip samples.

Wavenumber (cm^−1^)	Functional Group	Fraction [59,60]
3299	Symmetric and asymmetric stretching -O-H, stretching N-H	Hydroperoxides, amylose, amylopectin, and amide A
3009	Stretching C=C	Lipids
2923	Stretching asymmetric CH_2_	Lipids
2853	Stretching symmetric CH_2_	Lipids
1744	Stretching C=O	Triglycerides, phospholipids, and aldehydes
1559	Bending N-H	Amide II
1328	Bending N-H	Amide III
1034	Stretching C-O	Triglycerides

**Table 3:** Average values of wavenumber (cm^−1^) and absorbance of bands showing statistical differences (n ± SD; n = 3). Abs, absorbance; Wv, wavenumber.

Parameter	0 Days			6 Days			14 Days		
	Control	1.5 wt.%	3 wt.%	Control	1.5 wt.%	3 wt.%	Control	1.5 wt.%	3 wt.%
Wv at 1559 cm^−1^	1584 ± 3 ^a^	1563 ± 4 ^b^	1556 ± 3 ^c^	1553 ± 15 ^bc^	1557 ± 3 ^c^	1553 ± 2 ^c^	1568 ± 9 ^b^	1554 ± 1 ^c^	1542 ± 5 ^d^
Wv at 2853 cm^−1^	2855 ± 1 ^ab^	2856 ± 1 ^b^	2855 ± 1 ^ab^	2854 ± 1 ^ac^	2853 ± 1 ^c^	2853 ± 1 ^c^	2854 ± 1 ^ac^	2853 ± 1 ^c^	2851 ± 1 ^d^
Abs at 2923 cm^−1^	0.20 ± 0.01 ^a^	0.16 ± 0.02 ^b^	0.21 ± 0.01 ^a^	0.20 ± 0.02 ^a^	0.16 ± 0.02 ^b^	0.21 ± 0.01 ^a^	0.19 ± 0.01 ^a^	0.16 ± 0.01 ^b^	0.21 ± 0.01 ^a^
Wv at 2923 cm^−1^	2924 ± 1 ^a^	2827 ± 1 ^b^	2924 ± 1 ^a^	2923 ± 1 ^a^	2925 ± 2 ^a^	2922 ± 1 ^c^	2924 ± 1 ^a^	2920 ± 2 ^c^	2920 ± 5 ^abc^
Abs at 3009 cm^−1^	0.06 ± 0.01 ^a^	0.08 ± 0.02 ^a^	0.07 ± 0.02 ^a^	0.04 ± 0.02 ^a^	0.08 ± 0.03 ^b^	0.07 ± 0.03 ^b^	0.05 ± 0.01 ^a^	0.09 ± 0.01 ^b^	0.10 ± 0.02 ^b^
Abs at 3299 cm^−1^	0.08 ± 0.03 ^ab^	0.09 ± 0.02 ^a^	0.05 ± 0.06 ^b^	0.06 ± 0.03 ^b^	0.10 ± 0.02 ^a^	0.06 ± 0.02 ^b^	0.13 ± 0.02 ^c^	0.15 ± 0.02 ^c^	0.14 ± 0.01 ^c^
Wv at 3299 cm^−1^	3304 ± 1 ^a^	3303 ± 1 ^a^	3303 ± 3 ^a^	3302 ± 2 ^a^	3302 ± 2 ^a^	3296 ± 1 ^c^	3297 ± 1 ^c^	3294 ± 1 ^d^	3286 ± 1 ^e^

Different superscripts for each parameter within the same row indicate statistically significant different values (*p* < 0.05).

**Table 4:** Oleic, palmitic, linoleic, and linolenic fatty acid contents of chips (%), C18:2/C16:0 ratio and hexanal content (µg g^−1^) at 0, 6, and 14 days (n ± SD; n = 3).

Formulation	Parameter	Time (Days)		
		0	6	14
Control	Oleic	55.31 ± 1.29 ^ab^	56.50 ± 0.34 ^a^	54.21 ± 0.54 ^b^
1.5 wt.%		56.68 ± 0.34 ^a^	55.18 ± 0.87 ^a^	53.93 ± 0.47 ^b^
3.0 wt.%		56.41 ± 0.54 ^a^	53.35 ± 0.48 ^b^	52.62 ± 0.60 ^b^
Control	Palmitic	27.18 ± 1.30 ^a^	27.39 ± 0.63 ^a^	30.94 ± 0.46 ^b^
1.5 wt.%		28.90 ± 1.30 ^a^	31.91 ± 0.26 ^b^	31.51 ± 1.31 ^ab^
3.0 wt.%		27.57 ± 0.74 ^a^	34.14 ± 0.90 ^b^	32.88 ± 0.63 ^b^
Control	Linoleic	16.64 ± 0.75 ^a^	14.91 ± 0.76 ^a^	13.84 ± 0.12 ^b^
1.5 wt.%		13.80 ± 0.58 ^a^	13.58 ± 0.83 ^a^	11.67 ± 0.84 ^b^
3.0 wt.%		14.78 ± 1.29 ^a^	14.52 ± 0.74 ^a^	11.50 ± 0.45 ^b^
Control	Linolenic	1.20 ± 0.27 ^a^	1.20 ± 0.05 ^a^	1.02 ± 0.03 ^a^
1.5 wt.%		1.19 ± 0.13 ^a^	1.68 ± 0.08 ^a^	1.65 ± 0.12 ^a^
3.0 wt.%		1.24 ± 0.05 ^a^	1.68 ± 0.17 ^a^	0.97 ± 0.04 ^b^
Control	C18:2/C16:0	0.614 ± 0.052 ^a^	0.544 ± 0.017 ^a^	0.447 ± 0.006 ^a^
1.5 wt.%		0.479 ± 0.034 ^a^	0.426 ± 0.025 ^a^	0.371 ± 0.033 ^b^
3.0 wt.%		0.537 ± 0.060 ^a^	0.426 ± 0.032 ^b^	0.350 ± 0.009 ^c^
Control	Hexanal	525 ± 62 ^a^	521 ± 93 ^a^	494 ± 59 ^a^
1.5 wt.%		446 ± 73 ^a^	588 ± 33 ^a^	499 ± 72 ^a^
3.0 wt.%		458 ± 84 ^a^	405 ± 62 ^a^	465 ± 78 ^a^

Different superscripts for each parameter within the same row indicate statistically significant different values (*p* < 0.05).

## Data Availability

Data will be made available on request.
